# Mechanism of RNA‐Binding Protein ELAVL1 in Promoting Th2 Cell Differentiation Through Stabilizing CPA3 mRNA in Allergic Rhinitis

**DOI:** 10.1002/kjm2.70281

**Published:** 2026-09-02

**Authors:** Meng‐Ya Liu, Hong‐Bing Yao, Cheng Chen

**Affiliations:** ^1^ Department of Otolaryngology‐Head and Neck Surgery Children's Hospital of Chongqing Medical University Chongqing China; ^2^ National Clinical Research Center for Children and Adolescents' Health and Diseases Chongqing China; ^3^ Ministry of Education Key Laboratory of Child Development and Disorders Chongqing China; ^4^ Chongqing Key Laboratory of Pediatric Metabolism and Inflammatory Diseases Chongqing China

**Keywords:** allergic rhinitis, CPA3, ELAVL1, mRNA stability, Th2 differentiation

## Abstract

This study aimed to elucidate the role of the RNA‐binding protein embryonic lethality abnormal vision‐like protein 1 (ELAVL1) in the pathogenesis of allergic rhinitis (AR), specifically by investigating its potential to promote T helper 2 (Th2) cell differentiation by regulating the stability of carboxypeptidase A3 (CPA3) mRNA. Differentially expressed genes associated with AR were screened via bioinformatic analysis. Nasal mucosal tissues were collected from 42 children with AR and 42 healthy controls. CPA3 was significantly upregulated in the nasal mucosal tissues of AR patients. CD4^+^ T cells were isolated from the peripheral blood of healthy volunteers, polarized toward Th2 differentiation, and subjected to cell transfection experiments to examine the effect of CPA3 on Th2 differentiation. An ovalbumin (OVA)‐induced AR mouse model was established. Knockdown of CPA3 alleviated nasal symptoms (e.g., scratching and sneezing), attenuated pathological damage in the nasal mucosa (characterized by reduced edema and inflammatory cell infiltration), and suppressed Th2 immune responses (decreased interleukin‐4 and immunoglobulin E levels, reduced Th2 cell proportion, and downregulated GATA binding protein 3 expression) in AR mice. ELAVL1 bound to CPA3 mRNA and enhanced its stability, thereby upregulating CPA3 expression; a positive correlation was observed between their expression levels in AR. Overexpression of CPA3 reversed the alleviating effects of ELAVL1 knockdown on nasal symptoms and Th2 immune responses in AR mice. Taken together, these results demonstrate that targeting the ELAVL1/CPA3 axis may provide a novel therapeutic strategy for AR.

## 1 Introduction

Allergic rhinitis (AR) is a widespread immune‐related condition that frequently occurs in the global pediatric population, with epidemiological studies reporting prevalence rates ranging from 15% to 40%, and its prevalence continues to rise annually [1]. This disorder is characterized by excessive activation of T helper 2 (Th2)‐mediated immunity, the infiltration of eosinophils, and elevated levels of allergen‐specific immunoglobulin E (IgE). Clinically, it manifests as chronic inflammatory symptoms including nasal pruritus, recurrent sneezing, and nasal obstruction [2]. Notably, the immune system in children is still undergoing dynamic development, and the immaturity of the immunoregulatory network is closely associated with the risk of AR onset [3]. Within the complex immune microenvironment of AR, excessive activation of Th2 cells is considered a key factor disrupting immune balance [4]. Therefore, elucidating the functional regulatory mechanisms of Th2 cell differentiation in pediatric AR, particularly the protein interaction networks within local tissue microenvironments, is of urgent scientific significance for understanding disease pathogenesis and developing targeted interventions.

RNA‐binding proteins (RBPs) serve as crucial post‐transcriptional regulators that play a central role in gene expression regulation [5]. By binding to specific sequences or structures of RNA molecules, RBPs participate in multiple processes including transcription, splicing, transport, translation, and RNA stability control, thereby influencing protein expression and function [6, 7]. Recent studies have revealed that RBPs play important roles in the development, differentiation, and functional regulation of immune cells and are closely involved in various immune‐related diseases. For instance, in autoimmune diseases, infectious diseases, and tumor immune evasion, RBPs modulate the expression of key immune molecules, influencing immune cell differentiation and function, thereby contributing to disease pathogenesis [8, 9]. However, research on the role of RBPs in the pathogenesis of AR remains in its early stages. Further exploration of the functions and regulatory mechanisms of RBPs in AR may provide new insights and therapeutic targets for the treatment of this disease.

The embryonic lethal abnormal vision (ELAV) family represents a class of RBPs. Among its members, Embryonic lethal abnormal vision‐like protein 1 (ELAVL1), also referred to as Hu antigen R, is widely expressed in various tissues including the small intestine, spleen, and ovaries [10]. ELAVL1 has been shown to be upregulated in multiple cancer types, where it drives tumor initiation and progression, and is implicated in key oncogenic processes such as cell proliferation, migration, invasion, apoptosis, cell cycle regulation, chemoresistance, and radiation resistance [11, 12]. The protein regulates its downstream targets primarily through post‐transcriptional mechanisms, including the modulation of mRNA stability and translational efficiency [13]. More recently, ELAVL1 has been recognized as an m^6^A‐binding protein that regulates the stability and translation of target transcripts in an m^6^A‐dependent manner [14]. Additionally, emerging evidence indicates that ELAVL1 contributes to the modulation of antitumor immunity [15]. Furthermore, through m^6^A‐mediated mechanisms, it indirectly upregulates PD‐L1 expression by controlling downstream factors such as CMTM6 and MIR155HG [16]. Although previous studies have indicated that ELAVL1 can regulate Th2‐driven airway inflammation and thereby contribute to asthma [17, 18], it remains unclear whether it plays a similar role in AR, and what the specific downstream targets and molecular mechanisms are.

Carboxypeptidase A3 (CPA3) is a carboxypeptidase primarily expressed by mast cells and plays a significant role in allergic reactions [19]. CPA3 is not only involved in mast cell degranulation, releasing bioactive substances such as histamine and tryptase that trigger allergic symptoms, but also promotes allergic responses by regulating Th2 cell differentiation and cytokine secretion [20]. Studies have shown that CPA3 is highly expressed in the nasal mucosa and peripheral blood of AR patients, and its expression level is positively correlated with disease severity [21, 22]. However, the molecular mechanisms regulating CPA3 expression remain unclear, particularly the role of RBPs in controlling CPA3 mRNA stability.

Based on the above background, a scientific hypothesis is proposed: the RNA‐binding protein ELAVL1 may directly bind to and stabilize CPA3 mRNA, promoting CPA3 protein expression, thereby driving Th2 cell differentiation and ultimately contributing to the pathogenesis of AR. This study was designed to systematically elucidate the role and molecular mechanisms of ELAVL1 in AR through bioinformatic analyses, clinical sample testing, animal model construction, and molecular biology experiments. The findings are expected to reveal the critical role of the ELAVL1–CPA3 axis in regulating Th2 differentiation in AR, providing a new theoretical foundation and potential therapeutic target for the precise diagnosis and treatment of AR.

## 2 Materials and Methods

### 2.1 Bioinformatic Analysis

Differential gene expression analysis between groups was performed to identify differentially expressed genes (DEGs) based on quantitative expression results. The edgeR software package was used to perform differential analysis of gene expression between samples or groups. Filtered raw counts were normalized using the DESeq method, and DEGs were identified with screening thresholds set at |log_2_FC| > 1.5 and *p* < 0.05. Hierarchical clustering of DEGs was conducted using R/Bioconductor packages. To identify unique biological processes, cellular components, and molecular functions, Gene Ontology (GO) enrichment analysis was performed on significant DEGs. GO enrichment was carried out with clusterProfiler, and results were visualized using the ggplot2 package, with a significance threshold of *p* < 0.05. Differential gene correlation analysis was performed using dataset GSE19187 from the Gene Expression Omnibus database. Potential binding targets of ELAVL1 and CPA3 were predicted using starBase3.0.

### 2.2 Clinical Sample Collection

A total of 42 children with AR treated at Children's Hospital of Chongqing Medical University between January 2024 and March 2025 were selected as the case group, including 23 males and 19 females, aged 4 to 12 years (with a mean age of 7.59 ± 0.56 years). Allergen types included seasonal allergens (*n* = 30) and perennial allergens (*n* = 12). Additionally, 42 healthy children examined during the same period were selected as the control group, including 28 males and 14 females, aged 3 to 13 years (with a mean age of 7.62 ± 0.48 years). Nasal mucosal tissues were collected under direct visualization from the inferior turbinate surface of both AR patients and non‐AR healthy children using plastic scrapers. No significant differences were found in general characteristics between the two groups (*p* > 0.05). Inclusion criteria: (1) Patients who met the diagnostic criteria of the Chinese Guidelines for the Diagnosis and Treatment of Allergic Rhinitis, with positive skin prick test and serum specific IgE; (2) Patients diagnosed for the first time; (3) Patients whose guardians provided informed consent. Exclusion criteria: (1) Patients with infectious nasal diseases; (2) Patients with asthma; (3) Patients with sinusitis; (4) Patients with other concurrent infections; (5) Patients whose guardians declined to participate. Total RNA was extracted using the Trizol method from five randomly selected samples in each group and subjected to quantitative real‐time polymerase chain reaction (RT‐qPCR) analysis. The present study was approved by the Ethics Committee of Children's Hospital of Chongqing Medical University (Approval No. 202205CQ16) and written informed consent was provided by all patients' parents or guardians prior to the study start. All procedures were performed in accordance with the ethical standards of the Institutional Review Board and the Declaration of Helsinki, and its later amendments or comparable ethical standards.

### 2.3 CD4 ^+^ T Cell Isolation and Th2 Polarization

Peripheral blood was collected from healthy volunteers, and peripheral blood mononuclear cells (PBMCs) were separated using density gradient centrifugation. CD4 ^+^ T lymphocytes were subsequently purified from the PBMCs using magnetic‐activated cell sorting with a commercial CD4 ^+^ T Cell Isolation Kit (Miltenyi Biotech, Germany). To promote Th2 cell differentiation, the isolated cells were cultured for 7 days in Roswell Park Memorial Institute (RPMI) 1640 medium, supplemented with anti‐CD3 (3 μg/mL), anti‐CD28 (5 μg/mL), recombinant human interleukin‐4 (IL‐4, 20 ng/mL), and anti‐interferon‐γ (IFN‐γ, 20 μg/mL) [23]. In addition, ovalbumin (OVA, 1 μg/mL) was introduced to stimulate an inflammatory reaction in the CD4 ^+^ T cells. Every 2–3 days, the culture medium was replaced with fresh medium containing the same cytokine and antibody concentrations. All cellular samples used in this study were obtained from healthy donors.

### 2.4 Cell Culture

Isolated CD4 ^+^ T cells were cultured in RPMI 1640 medium (Sigma‐Aldrich, USA) supplemented with penicillin–streptomycin, glutamine, and fetal bovine serum. The medium was replaced every 2–3 days. Cells were maintained at 37°C in a 5% CO_2_ atmosphere. The cell concentration was adjusted to 1 × 10^6^/mL for subsequent experiments.

### 2.5 Cell Transfection

Short hairpin RNAs (shRNAs) targeting CPA3 and ELAVL1 (sh‐CPA3, sh‐ELAVL1), overexpression plasmid pcDNA3.1‐CPA3, and their corresponding negative controls (sh‐NC, pcDNA3.1‐NC) were designed and synthesized by GenePharma (China). Transfection was performed using Lipofectamine 3000 (Invitrogen, USA) according to the manufacturer's instructions. Transfected CD4^+^ T cells were collected 48 h post‐transfection for further experiments. Th2 polarization was induced 24 h after transfection.

### 2.6 Identification of IL‐4–Expressing Cells

Sorted CD4^+^ T cells were adjusted to a density of 1 × 10^6^/mL and fixed in 2% paraformaldehyde for 2 h at room temperature. Cells were permeabilized with 0.5% saponin for 30 min. After washing with phosphate‐buffered saline (PBS), the cells were stained with a fluorescein isothiocyanate‐conjugated anti‐IL‐4 antibody for 1 h. The stained cells were analyzed using a FACSCanto II flow cytometer (BD Biosciences, USA).

### 2.7 AR Modeling and Intervention

Seventy‐five female BALB/c mice were purchased from Apex Experimental Animal Breeding Co. Ltd. (China). Prior to modeling, the mice were acclimated for 1 week in individual cages under controlled conditions: room temperature 22.5°C ± 2.5°C, relative humidity 50% ± 10%, and an air exchange rate of 10–15 times per hour. All animal experiments were conducted in compliance with the ARRIVE guidelines and in accordance with the National Institutes of Health Guide for the Care and Use of Laboratory Animals. The experiments were approved by the Institutional Animal Care and Use Committee of Children's Hospital of Chongqing Medical University (Approval No. 202212‐CQ06). After 1 week of acclimation, the animals were randomly divided into groups using a random number table. Throughout the entire experiment, including animal grouping, intervention administration, behavioral tests, and histopathological analyses, all procedures were performed by independent investigators who were blinded to the group assignments.

An OVA‐induced AR mouse model was established over a two‐week period. On days 0, 7, and 14, mice in the AR group were intraperitoneally injected with a mixture of 25 μg OVA and 2 mg aluminum hydroxide (Al(OH)_3_) in PBS, in a total volume of 250 μL. From day 21 to day 30, the mice received daily intranasal challenges with 20 μL of 3% OVA solution. The control group (*n* = 5) received the same volume of PBS following identical procedures. The AR group was further randomly divided into the model group (OVA), sh‐NC group, sh‐CPA3 group, sh‐ELAVL1 group, pcDNA3.1‐NC group, pcDNA3.1‐CPA3 group, and sh‐ELAVL1 + pcDNA3.1‐CPA3 group (10 mice per group). Mice in the model group received intraperitoneal injections and intranasal instillations of normal saline at equivalent volumes. On days 0, 7, and 14, intranasal interventions of sh‐NC, sh‐CPA3, sh‐ELAVL1, pcDNA3.1‐NC, or pcDNA3.1‐CPA3 were performed, with 20 μL (10 μL per nostril) administered each time. Before intranasal administration of shRNA or plasmid, mice were anesthetized using isoflurane, and the solution was instilled slowly to ensure proper delivery. After successful modeling and treatment, mice were anesthetized with 0.6% sodium pentobarbital, and blood was collected from the retro‐orbital sinus prior to euthanasia.

### 2.8 Evaluation of Nasal Allergic Symptoms in Mice

Nasal allergic‐like symptoms were evaluated by counting the number of sneezing and nasal rubbing events following AR induction. Mice were placed individually in plastic animal cages (35 × 20 × 30 cm), and the numbers of sneezing and rubbing episodes were recorded over a 10‐min period.

### 2.9 Enzyme‐Linked Immunosorbent Assay (ELISA)

Nasal mucosa and blood samples were collected from the nostrils and the retro‐orbital plexus 24 h after the final AR challenge. Blood samples were incubated to clot at room temperature for 30 min and then centrifuged at 3000 *g* for 15 min at room temperature. Serum was collected, and both nasal mucosa and serum samples were stored at −80°C until further analysis. The concentrations of OVA‐specific IgE, IFN‐γ, and IL‐4 were measured using corresponding ELISA kits.

### 2.10 Flow Cytometry

Harvested cells were resuspended in medium containing 10% fetal bovine serum. After 72 h of culture, designated cells were collected and resuspended. Cell suspensions were stimulated with Phorbol 12‐myristate 13‐acetate (25 ng/mL), ionomycin (1 μg/mL), and GolgiStop (5 ng/mL) for 4 h at 37°C. Th1 and Th2 cell subsets were analyzed based on intracellular cytokine expression of IFN‐γ and IL‐4 in CD3^+^CD4^+^ T cells.

### 2.11 Hematoxylin–Eosin (H&E) Staining

Nasal mucosal tissues from mice were fixed in 4% paraformaldehyde for 24 h, dehydrated, embedded in paraffin, and sectioned coronally at a thickness of 4 μm. H&E staining (Beyotime, China) was performed in accordance with the manufacturer's instructions. Sections were stained with hematoxylin for 15 min, differentiated in 1% hydrochloric acid alcohol for 10 s, rinsed with 2% sodium bicarbonate (Beyotime) for 10 s, and then counterstained with eosin for 3 min. After dehydration through a graded alcohol series, the sections were cleared in xylene (214,736; Sigma‐Aldrich) and mounted with neutral resin. Pathological changes in the nasal mucosa were observed under a microscope (Olympus BX53, Japan).

### 2.12 Immunohistochemistry (IHC)

Nasal mucosal tissues were fixed in 4% paraformaldehyde, dehydrated through an ethanol series, and embedded in paraffin. Sections were deparaffinized, rehydrated, treated with 3% H_2_O_2_ to block endogenous peroxidase activity, and antigen retrieval was performed by boiling in sodium citrate buffer for 30 min. Permeabilization was carried out with 0.5% Triton X‐100, followed by blocking with 1% bovine serum albumin. Tissue sections were incubated overnight at 4°C with primary antibodies against GATA binding protein 3 (GATA3; ab282110, Abcam, UK) and T‐box expressed in T cells (T‐bet; ab307193, Abcam) at a dilution of 1:500. Subsequently, the sections were incubated for 1 h at room temperature with horseradish peroxidase (HRP)‐conjugated secondary antibodies: Goat Anti‐Rabbit IgG (H + L) (Thermo Fisher Scientific, USA; 31,460) and Goat Anti‐Mouse IgG (H + L) (7076S, Cell Signaling Technology, USA), diluted 1:1000. Following incubation with an avidin‐biotin‐peroxidase complex, color development was performed using 3,3′‐diaminobenzidine tetrahydrochloride substrate (ZSGB‐BIO, China). The sections were then counterstained with hematoxylin to visualize nuclei. Finally, slides were observed under a microscope (Leica, Germany).

### 2.13 Western Blot

Nasal mucosal tissues and cells were homogenized in radioimmunoprecipitation assay (RIPA) buffer (Merck Millipore, 20–188) containing protease (Beyotime) and phosphatase inhibitors (Beyotime). The homogenates were centrifuged at 12,000 *g* for 10 min at 4°C to collect the supernatant. Protein concentration was determined using a bicinchoninic acid kit (Beyotime). Proteins (30 μg per lane) were separated by sodium dodecyl sulfate‐polyacrylamide gel electrophoresis (Beyotime) and transferred onto polyvinylidene fluoride membranes (Millipore, USA) via electroblotting. The membranes were blocked with 5% skim milk for 2 h and then incubated overnight at 4°C with primary antibodies against ELAVL1 (ab170193, Abcam), CPA3 (ab158188, Abcam), GATA3 (ab282110, Abcam), T‐bet (ab307193, Abcam), and glyceraldehyde‐3‐phosphate dehydrogenase (GAPDH; ab37168, Abcam). Subsequently, the membranes were incubated with an HRP‐conjugated anti‐rabbit or anti‐mouse secondary antibody (1:500; ab288151, ab6789, Abcam) at 37°C for 1 h. GAPDH was used as the internal control. Protein bands were visualized using an enhanced chemiluminescence detection kit (Vazyme, China; E411‐04) and imaged on a FluorChem M system.

### 2.14 RT‐qPCR

Total RNA was extracted from tissues and cells using Trizol reagent (Invitrogen, USA), and RNA was synthesized into cDNA using PrimeScript RT kit (Takara, Japan) according to the manufacturer's protocol and stored at −20°C. Subsequently, quantitative real‐time PCR in triplicate was performed using TB Green Premix Ex Taq (RR820A, Takara, Japan) in the ROCHE LightCycler480 system (Roche Diagnostics GmbH, Germany). β‐actin was used as the internal reference gene, and the relative mRNA expression level was calculated by the 2^−ΔΔCt^ method. The primers are shown in Table 1.

**TABLE 1:** Primers sequences for RT‐qPCR.

Genes	Primer sequence (5′–3′)
UTY	F: 5′‐GGAGAAGGTGTTGCTGTGGA‐3′ R: 5′‐AGCCAATGGCAATGTGGGTA‐3′
HBA2	F: 5′‐TGCTGACCTCCAAATACCGT‐3′ R: 5′‐CCTCCATTGTTGGCACATTCC‐3′
POSTN	F: 5′‐GCTATTCTGACGCCTCAAAACT‐3′ R: 5′‐AGCCTCATTACTCGGTGCAAA‐3′
CPA3	F: 5′‐ATTCACGCACGAGAATGGGT‐3′ R: 5′‐ATGGTCTCTCTGCACGTTGG‐3′
SCT2	F: 5′‐TAACTGGTGGTCCTGGATGC‐3′ R: 5′‐TTTGGTGTCGCTCGTCTCAG‐3′
GAPDH	F: 5′‐AGGTCGGTGTGAACGGATTTG‐3′ R: 5′‐GGGGTCGTTGATGGCAACA‐3′

### 2.15 RNA Immunoprecipitation (RIP) Assay

RIP assays were performed using a commercial RIP kit (Geneseed, China) following the manufacturer's instructions. Briefly, transfected HEK293 cells were initially lysed in RIPA buffer supplemented with protease and phosphatase inhibitors (Beyotime). Cell lysates were incubated overnight at 4°C with magnetic beads conjugated with either an anti‐ELAVL1 antibody or control IgG in RIP buffer. Co‐precipitated RNA was then extracted through proteinase K treatment. The isolated RNA was subsequently analyzed by RT‐qPCR.

### 2.16 RNA Pull‐Down Assay for Protein–RNA Interaction

RNA pull‐down assays were performed using an RNA pull‐down kit (Thermo Fisher Scientific) to identify proteins that bind to CPA3 RNA. Biotin‐labeled CPA3 RNA probes and negative control probes were incubated with streptavidin‐coated magnetic beads at room temperature for 30 min. After discarding the supernatant, HEK293 cell lysates were added to the bead‐probe complexes and incubated at 4°C for 1 h to allow protein binding. Bound proteins were eluted using elution buffer and analyzed by Western blot. ELAVL1 protein was detected using an anti‐ELAVL1 primary antibody (ab170193, Abcam; 1:1000 dilution).

### 2.17 Immunofluorescence

HEK293 cells were seeded onto coverslips and cultured until they reached 60%–70% confluence. The medium was then removed, and the cells were washed with PBS, fixed with 4% paraformaldehyde for 20 min at room temperature, and permeabilized with 0.5% Triton X‐100 for 15 min at room temperature. Subsequently, the cells were blocked with 5% bovine serum albumin for 1 h at room temperature. After blocking, primary antibodies, ab170193 (Abcam) and anti‐CPA3 (DF14338, Affinity), were applied and incubated overnight at 4°C. The next day, the cells were incubated with anti‐rabbit or anti‐mouse secondary antibodies (ab150113, ab150084, Abcam) for 1 h at room temperature in the dark. Following incubation, nuclei were counterstained with DAPI for 5 min in the dark. Finally, the coverslips were mounted with an anti‐fade mounting medium, and images were acquired using a laser scanning confocal microscope (Zeiss, Germany).

### 2.18 Actinomycin D Assay for mRNA Stability

To evaluate the effect of ELAVL1 on CPA3 mRNA stability, transfected HEK293 cells were treated with 5 μg/mL actinomycin D (SBR00013, Sigma‐Aldrich). After incubation for the indicated time periods, cells were collected at 0, 2, 4, 6, and 8 h post‐treatment. RNA was extracted from each sample, and CPA3 expression levels were analyzed using RT‐qPCR. GAPDH was used as an internal reference for normalization.

### 2.19 Statistical Analysis

Data were analyzed using GraphPad Prism software and are presented as mean ± standard deviation (SD). Comparisons between two groups were performed using an unpaired two‐tailed Student's *t*‐test, while comparisons among multiple groups were analyzed using one‐way or two‐way analysis of variance (ANOVA). A *p*‐value < 0.05 was considered statistically significant. All experiments were performed in three independent replicates.

## 3 Results

### 3.1 CPA3 Expression Is Upregulated in the Nasal Mucosa of AR Patients

**FIGURE 1:**
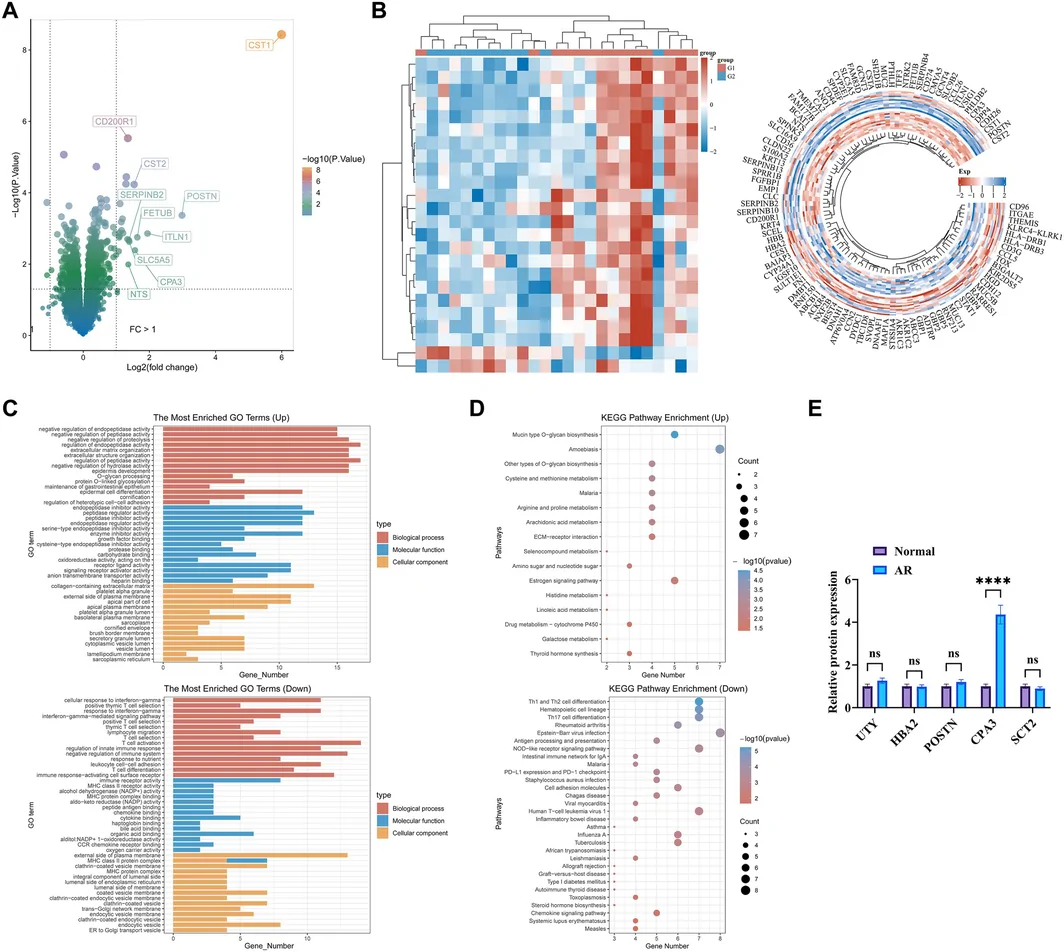
CPA3 is upregulated in AR. (A) Volcano plot showing DEGs. (B) Heatmap displaying the top 10 upregulated and downregulated mRNAs. (C) GO enrichment analysis. (D) KEGG pathway enrichment analysis. (E) mRNA expression levels of UTY, HBA2, POSTN, CPA3, and SCT2 in nasal mucosal tissues from healthy controls and AR patients, as detected by RT‐qPCR. Error bars represent at least three independent experiments; data are presented as mean ± SD. Comparisons were made using Student's *t*‐test and one‐way ANOVA. ****p < 0.0001; ns, not significant.

To profile the transcriptome of allergic rhinitis, RNA sequencing was performed on nasal mucosal tissues obtained from 14 AR patients and 11 healthy donors. A threshold of |log_2_FC| > 1.5 and *p* < 0.05 was applied, identifying 24 differentially expressed mRNAs, among which 22 were upregulated and 2 downregulated (Figure 1A). A hierarchical clustering heatmap was constructed to visualize the top five upregulated and downregulated transcripts (Figure 1B). Notably, POSTN, CPA3, and SCT2 mRNAs were significantly elevated in AR tissues (*p* < 0.05), while UTY and HBA2 mRNAs were markedly reduced (*p* < 0.05), suggesting a potential involvement in AR pathogenesis. Next, to explore the functional relevance of these DEGs, GO, and Kyoto Encyclopedia of Genes and Genomes (KEGG) pathway analyses were conducted. GO analysis revealed significant enrichment in immune‐related biological processes, such as T cell activation, immune system regulation, and cell surface receptor‐mediated immune responses (Figure 1C). KEGG analysis showed that these DEGs were notably enriched in immune‐related pathways, including the NOD‐like receptor signaling pathway, cytokine–cytokine receptor interactions, and neuroactive ligand–receptor interactions (Figure 1D). To experimentally verify the RNA‐seq results, RT‐qPCR was carried out on five additional independent sample pairs. The results confirmed that the expression of UTY and HBA2 was lower in AR tissues, whereas POSTN, CPA3, and SCT2 were higher, with CPA3 exhibiting statistically significant upregulation (*n* = 5, *p* < 0.0001; Figure 1E). Based on these observations, subsequent research was directed toward elucidating the molecular mechanisms through which CPA3 influences AR.

### 3.2 Knockdown of CPA3 Alleviates Nasal Symptoms in AR Mice

**FIGURE 2:**
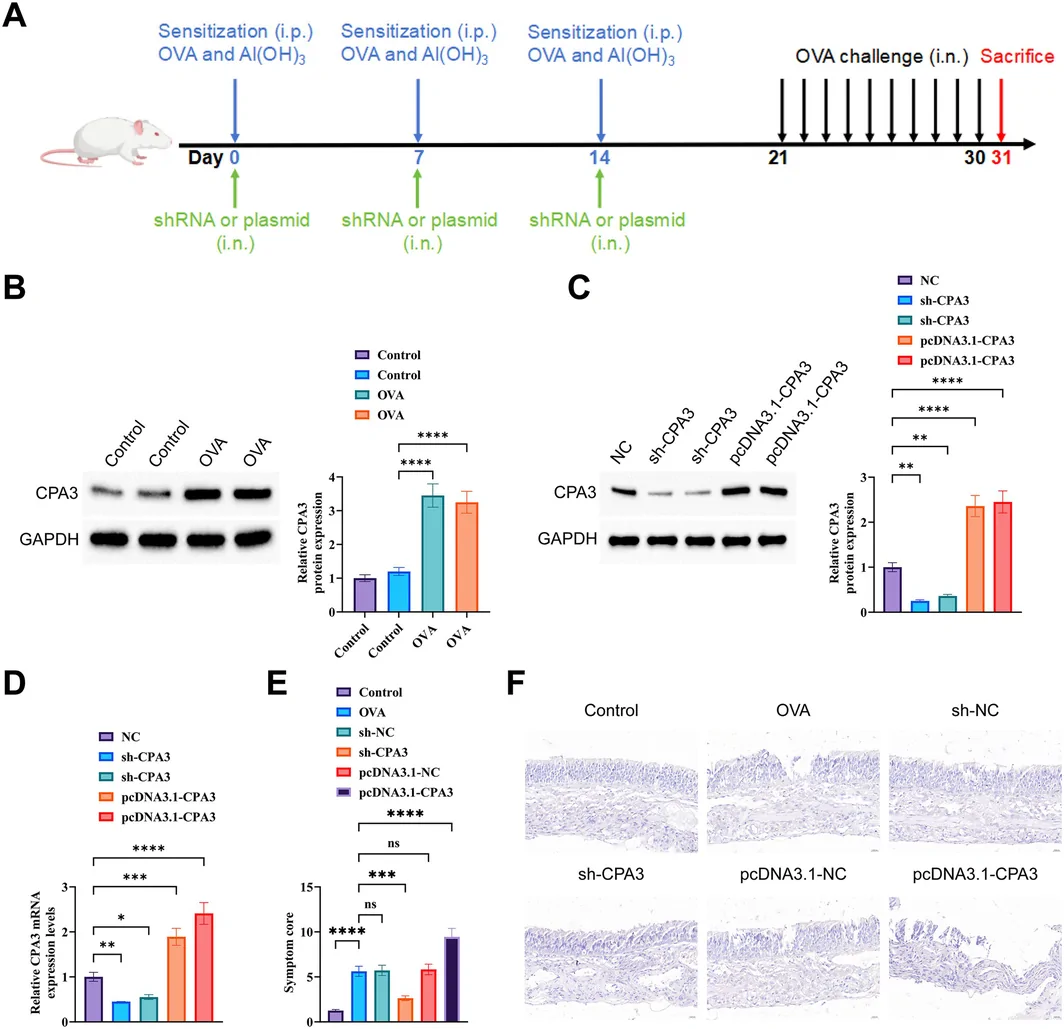
Knockdown of CPA3 alleviates nasal symptoms in AR mice. (A) Schematic diagram of the OVA‐induced modeling. (B) Protein expression of CPA3 in mouse nasal mucosal tissues, as detected by Western blot. (C) After intranasal administration of sh‐CPA3 or pcDNA3.1‐CPA3, the mRNA expression level of CPA3 in mouse nasal mucosal tissues was detected by RT‐qPCR. (D) After intranasal administration of sh‐CPA3 or pcDNA3.1‐CPA3, the protein expression of CPA3 in mouse nasal mucosal tissues was detected by Western blot. (E) Behavioral observations of mice. (F) Pathological examination of nasal mucosal tissues from each group by H&E staining. Error bars represent at least three independent experiments; data are presented as mean ± SD. Comparisons were made using Student's *t*‐test and one‐way ANOVA. *p < 0.05, **p < 0.01, ***p < 0.001, ****p < 0.0001; ns, not significant.

Previous studies have indicated that CPA3 plays an indispensable role in various diseases, though its function in AR remains unclear. To investigate the role of CPA3 in the pathogenesis of AR, an OVA‐induced sensitization and challenge protocol was used to establish an AR mouse model (Figure 2A). Western blot analysis revealed that CPA3 protein expression was significantly elevated in the nasal mucosal tissues of AR mice compared with the control group (Figure 2B), suggesting a potential role of CPA3 in AR development. To further examine the functional impact of CPA3, plasmids carrying shRNA targeting CPA3 (sh‐CPA3) or a CPA3 overexpression plasmid (pcDNA3.1‐CPA3) were intranasally administered to knock down or overexpress CPA3, respectively. The efficiency of these interventions was evaluated using RT‐qPCR and Western blot. As shown in Figure 2C, compared with the control group, CPA3 expression was significantly decreased in the sh‐CPA3 group and increased in the pcDNA3.1‐CPA3 group at the mRNA level. Consistent results were obtained at the protein level (Figure 2D). Behavioral tests indicated that AR mice showed significantly more scratching and sneezing episodes compared with controls. These symptoms were alleviated in the sh‐CPA3 group but exacerbated in the pcDNA3.1‐CPA3 group (Figure 2E). H&E staining (Figure 2F) revealed typical pathological damage in the nasal mucosa of AR mice, including ciliary damage, epithelial detachment, submucosal edema, inflammatory cell infiltration, and glandular hyperplasia. These pathological changes were markedly attenuated in the sh‐CPA3 group, with reduced edema and inflammatory cell infiltration. In contrast, the pcDNA3.1‐CPA3 group showed aggravated damage, including more severe mucosal swelling, ciliary loss, glandular hyperplasia, and inflammatory infiltration compared with the AR group. These results demonstrate that knockdown of CPA3 alleviates nasal symptoms in AR mice.

### 3.3 CPA3 Exacerbates AR Progression by Enhancing Th2 Immune Responses

**FIGURE 3:**
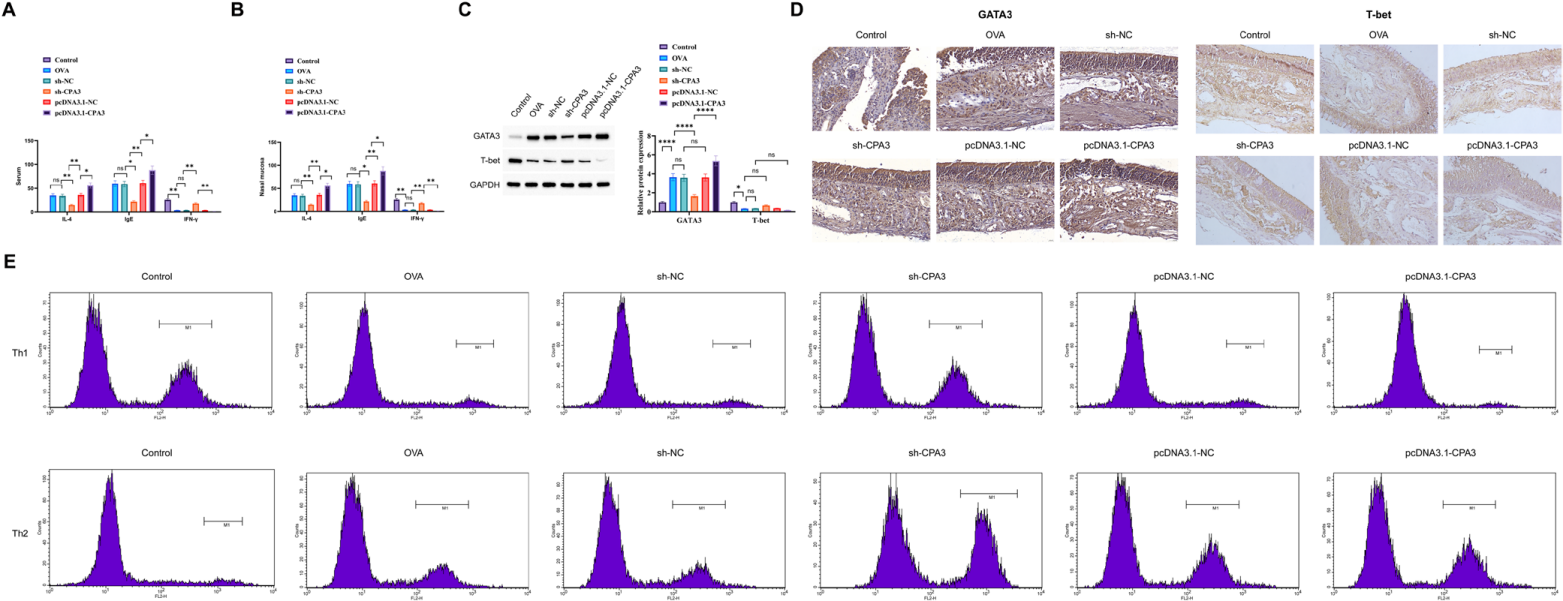
CPA3 exacerbates AR progression by enhancing Th2 immune responses. (A) Serum levels of OVA‐specific IgE, IL‐4, and IFN‐γ, as detected by ELISA. (B) Levels of OVA‐specific IgE, IL‐4, and IFN‐γ in nasal mucosal tissues, as detected by ELISA. (C) Protein expression of T‐bet and GATA3 in mouse nasal mucosal tissues, as detected by Western blot. (D) Expression of T‐bet and GATA3 in mouse nasal mucosal tissues, as detected by IHC. (E) Proportion of Th1/Th2 cells, as detected by flow cytometry. Error bars represent at least three independent experiments; data are presented as mean ± SD. Comparisons were made using Student's *t*‐test and one‐way ANOVA. ***p < 0.001, ****p < 0.0001; ns, not significant.

To investigate whether CPA3 influences the secretion of allergic mediators, ELISA was used to quantify the concentrations of IL‐4, IFN‐γ, and OVA‐specific IgE in both serum and nasal mucosal tissues. Elevated levels of IgE and IL‐4, along with reduced IFN‐γ, were observed in AR mice compared with controls. In mice treated with sh‐CPA3, IgE, and IL‐4 levels were noticeably decreased, while IFN‐γ expression was increased relative to the AR group. Conversely, animals transfected with pcDNA3.1‐CPA3 exhibited further rises in IgE and IL‐4 and a more pronounced reduction in IFN‐γ (Figure 3A,B). Given that the transcription factors T‐bet and GATA3 play critical roles in driving the differentiation of naïve T cells into IFN‐γ‐producing Th1 and IL‐4‐secreting Th2 cells, respectively [24], the effect of CPA3 on their protein expression was subsequently evaluated. Western blot analysis revealed that GATA3 expression was upregulated and T‐bet downregulated in AR mice compared with controls. These trends were reversed following sh‐CPA3 treatment, whereas pcDNA3.1‐CPA3 transfection further enhanced GATA3 and suppressed T‐bet expression (Figure 3C). Consistent results were obtained through IHC staining and optical quantification, which indicated increased GATA3 and decreased T‐bet in AR tissues—effects that were attenuated with sh‐CPA3 and aggravated with CPA3 overexpression (Figure 3D). Furthermore, flow cytometry was employed to assess Th1/Th2 polarization in nasal mucosa. AR mice displayed an increased proportion of IL‐4‐producing Th2 cells (BV421⁺) and a decreased frequency of IFN‐γ‐producing Th1 cells (PE‐CY7⁺). Knockdown of CPA3 reduced Th2 and increased Th1 populations, while CPA3 overexpression further promoted Th2 polarization and inhibited Th1 differentiation (Figure 3E). Collectively, these findings demonstrate that CPA3 aggravates AR by promoting Th2 immune responses.

### 3.4 Knockdown of CPA3 Inhibits OVA‐Induced Th2 Differentiation of CD4^+^ T Cells

**FIGURE 4:**
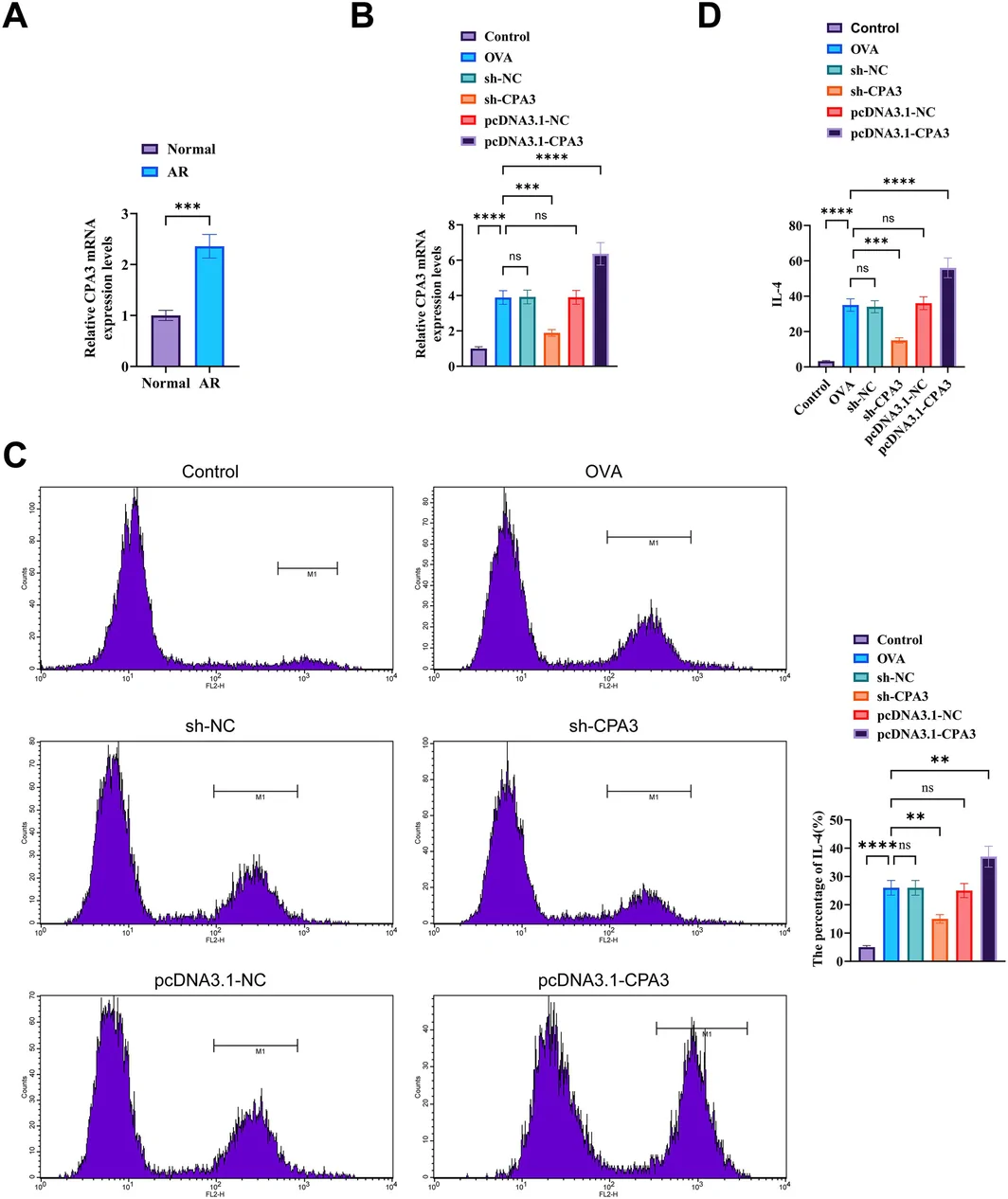
Knockdown of CPA3 inhibits OVA‐induced Th2 differentiation of CD4^+^ T Cells. (A) mRNA expression level of CPA3 in nasal mucosal tissue samples, as detected by RT‐qPCR. (B) mRNA expression level of CPA3 in OVA‐induced CD4^+^ T cells, as quantified by RT‐qPCR. (C) Percentage of IL‐4‐expressing cells, as determined by flow cytometry. (D) IL‐4 level in the supernatant of OVA‐induced CD4^+^ T cells, as detected by ELISA. Error bars represent at least three independent experiments; data are presented as mean ± SD. Comparisons were made using Student's *t*‐test and one‐way ANOVA. *p < 0.05, **p < 0.01, ***p < 0.001, ****p < 0.0001; ns, not significant.

To investigate the role of CPA3 in AR, its expression levels were assessed by RT‐qPCR in nasal mucosal tissues collected from AR patients and healthy controls. The results revealed a significant upregulation of CPA3 in AR samples compared to normal tissues (Figure 4A). Before OVA treatment and Th2‐polarizing stimulation, CD4^+^ T cells were transfected with either sh‐CPA3 or a CPA3 overexpression plasmid (pcDNA3.1‐CPA3). After transfection and OVA induction, RT‐qPCR analysis showed that OVA stimulation significantly increased CPA3 expression. Transfection with sh‐CPA3 effectively reduced CPA3 levels, whereas expression of pcDNA3.1‐CPA3 led to a further increase compared to the OVA‐treated group (Figure 4B). Flow cytometry was used to quantify the proportion of IL‐4‐producing cells. Silencing of CPA3 resulted in a significant decrease in IL‐4‐positive cells, whereas CPA3 overexpression significantly increased this population (Figure 4C). Consistent with these findings, ELISA results showed that sh‐CPA3 transfection reduced IL‐4 secretion in the supernatant of OVA‐induced CD4^+^ T cells, while pcDNA3.1‐CPA3 transfection significantly enhanced IL‐4 production (Figure 4D). Collectively, these results suggest that CPA3 knockdown attenuates OVA‐driven Th2 differentiation in CD4^+^ T cells.

### 3.5 ELAVL1 Binds to CPA3 mRNA and Upregulates Its Expression by Enhancing mRNA Stability

**FIGURE 5:**
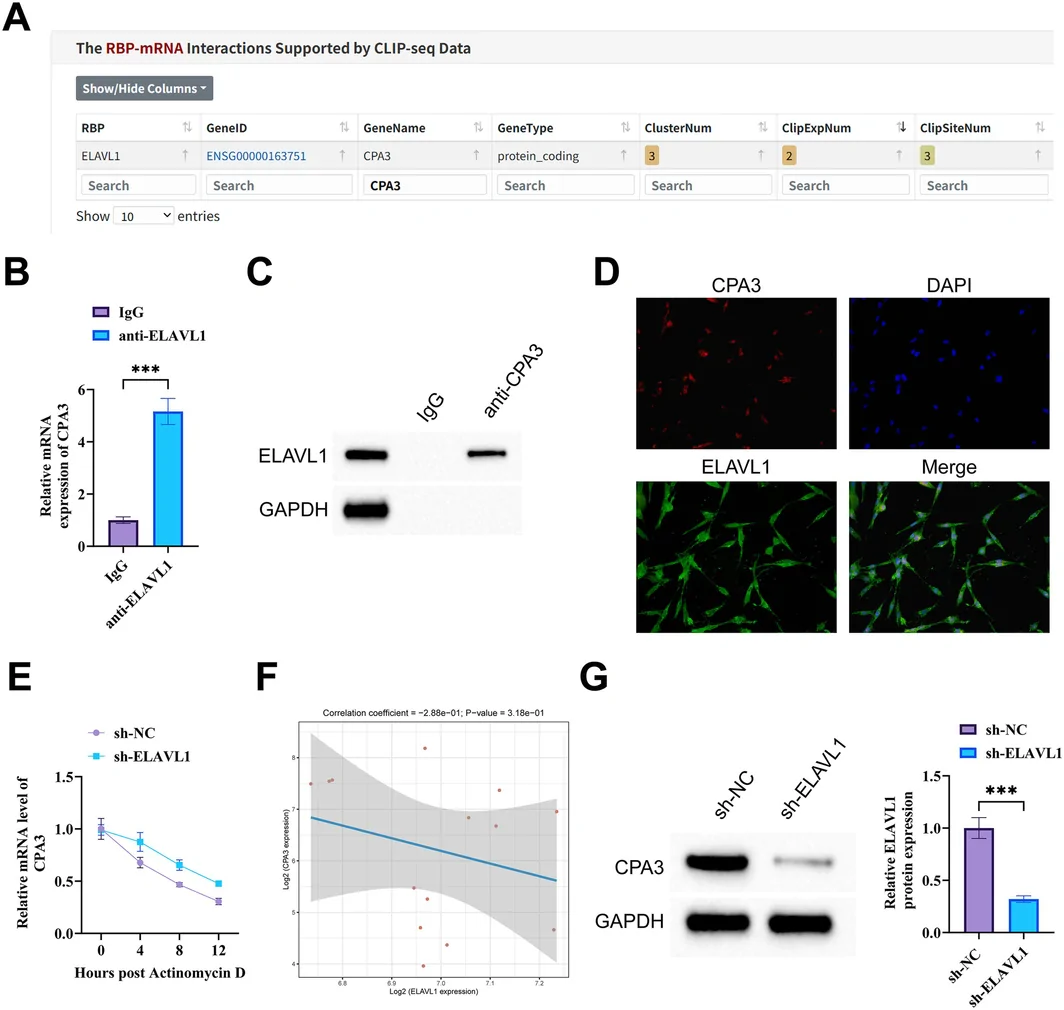
ELAVL1 binds to CPA3 mRNA and upregulates its expression by enhancing mRNA stability. (A) Predicted binding sites of ELAVL1 on CPA3 mRNA by StarBase 3.0. (B) Enrichment of CPA3 mRNA by ELAVL1, as assessed by RIP assay. (C) Interaction between ELAVL1 and CPA3, as confirmed by RNA pull‐down assay. (D) Co‐localization of ELAVL1 and CPA3, as detected by immunofluorescence. (E) Stability of CPA3 mRNA verified by actinomycin D assay to measure its half‐life. (F) Correlation analysis of the GSE19187 dataset showing a positive correlation between ELAVL1 and CPA3 gene expression. (G) Protein expression of CPA3 after ELAVL1 knockdown, as detected by Western blot. Error bars represent at least three independent experiments; data are presented as mean ± SD. Comparisons were made using Student's *t*‐test and one‐way ANOVA. ***p < 0.001.

To further investigate the role of CPA3 in AR progression, potential RNA‐binding partners for CPA3 were predicted using the starBase3.0 database. The results indicated that ELAVL1 may bind to multiple exonic regions of CPA3 mRNA (Figure 5A). RIP assays confirmed significant enrichment of CPA3 mRNA in ELAVL1 antibody immunoprecipitates compared to IgG controls (Figure 5B). RNA pull‐down assays further demonstrated a direct interaction between ELAVL1 and CPA3 (Figure 5C). Additionally, immunofluorescence assays revealed co‐localization of ELAVL1 and CPA3 proteins (Figure 5D). Actinomycin D treatment showed that knockdown of ELAVL1 reduced the stability of CPA3 mRNA (Figure 5E). Correlation analysis using the GSE19187 dataset indicated a positive relationship between ELAVL1 and CPA3 expression (*p* < 0.05, *R* = 0.53) (Figure 5F). Moreover, knockdown of ELAVL1 decreased CPA3 protein levels (Figure 5G). These findings demonstrate that ELAVL1 enhances the stability of CPA3 mRNA and promotes its expression.

### 3.6 Overexpression of CPA3 Reverses the Alleviation of Nasal Symptoms in AR Mice Induced by ELAVL1 Knockdown

**FIGURE 6:**
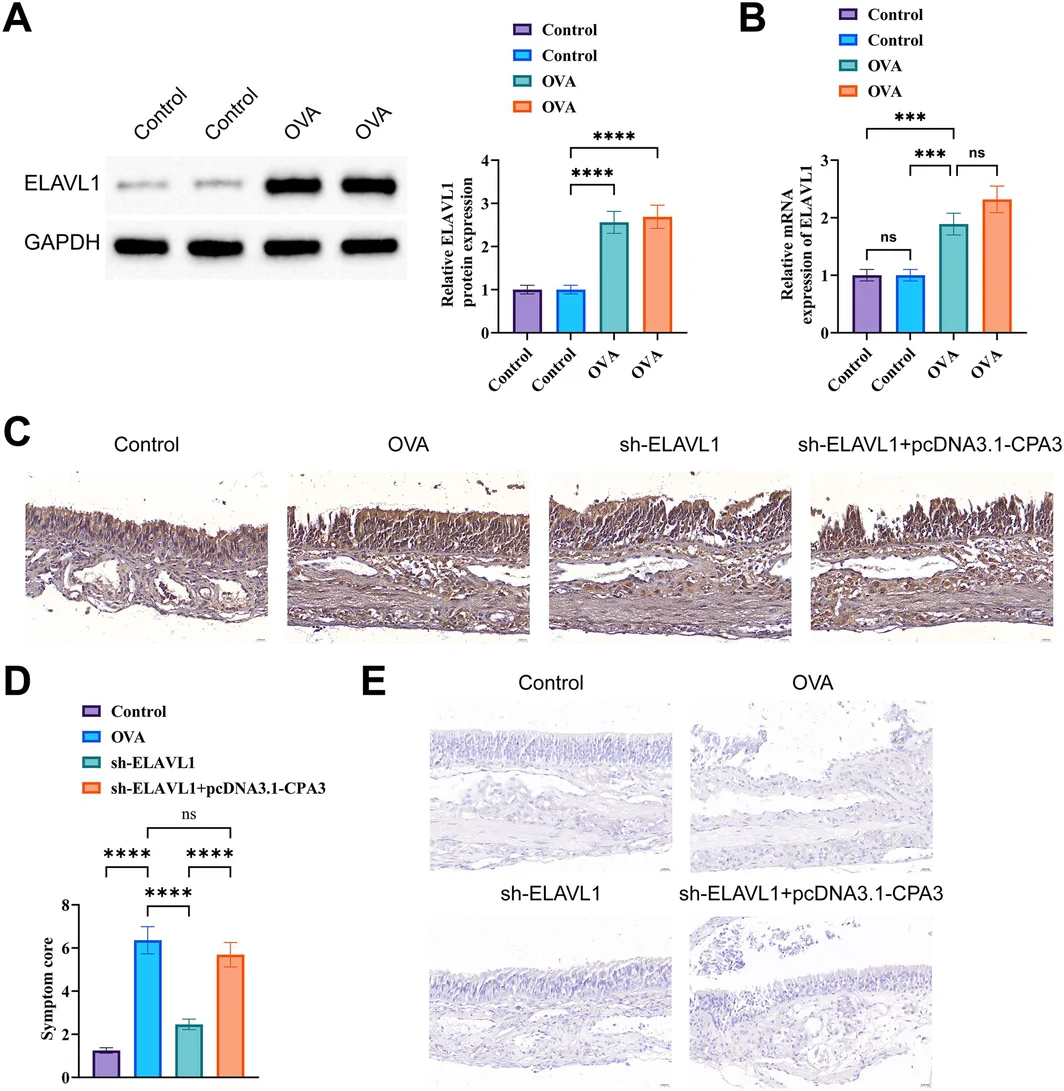
Overexpression of CPA3 reverses the alleviation of nasal symptoms in AR mice induced by ELAVL1 knockdown. (A) Protein expression of ELAVL1 in mouse nasal mucosal tissues, as detected by Western blot. (B) mRNA expression of ELAVL1 in mouse nasal mucosal tissues, as detected by RT‐qPCR. (C) Expression of CPA3 in mouse nasal mucosal tissues, as detected by IHC. (D) Behavioral observations of mice. (E) Pathological changes in mouse nasal mucosal tissues observed by H&E staining. Error bars represent at least three independent experiments; data are presented as mean ± SD. Comparisons were made using Student's *t*‐test and one‐way ANOVA. ***p < 0.001, ****p < 0.0001; ns, not significant.

The above results indicate that ELAVL1 interacts with and promotes the expression of CPA3. To clarify the role of ELAVL1‐mediated regulation of CPA3 in AR, the expression of ELAVL1 in nasal mucosal tissues of mouse models was first examined by Western blot. The results showed that ELAVL1 expression was significantly increased in the OVA group compared to the control group (Figure 6A). RT‐qPCR results were consistent with the Western blot data (Figure 6B). Additionally, IHC revealed elevated CPA3 expression in the nasal mucosa of OVA‐induced mice, which was reduced upon ELAVL1 knockdown, and overexpression of CPA3 further increased CPA3 protein levels in the nasal mucosa of OVA‐induced mice (Figure 6C). Behavioral tests indicated that overexpression of CPA3 reversed the alleviation of nasal scratching and sneezing frequencies induced by ELAVL1 knockdown, leading to a significant increase in both parameters (Figure 6D). H&E staining demonstrated that CPA3 overexpression counteracted the protective effects of ELAVL1 knockdown on nasal mucosal tissue injury, as shown by impaired cilia, epithelial damage and detachment, significant submucosal edema, extensive infiltration of eosinophils and other inflammatory cells, and noticeable glandular hyperplasia (Figure 6E). These findings indicate that ELAVL1 contributes to the pathogenesis of AR by promoting CPA3 expression.

### 3.7 Overexpression of CPA3 Reverses the Inhibitory Effect of ELAVL1 Knockdown on Th2 Differentiation

**FIGURE 7:**
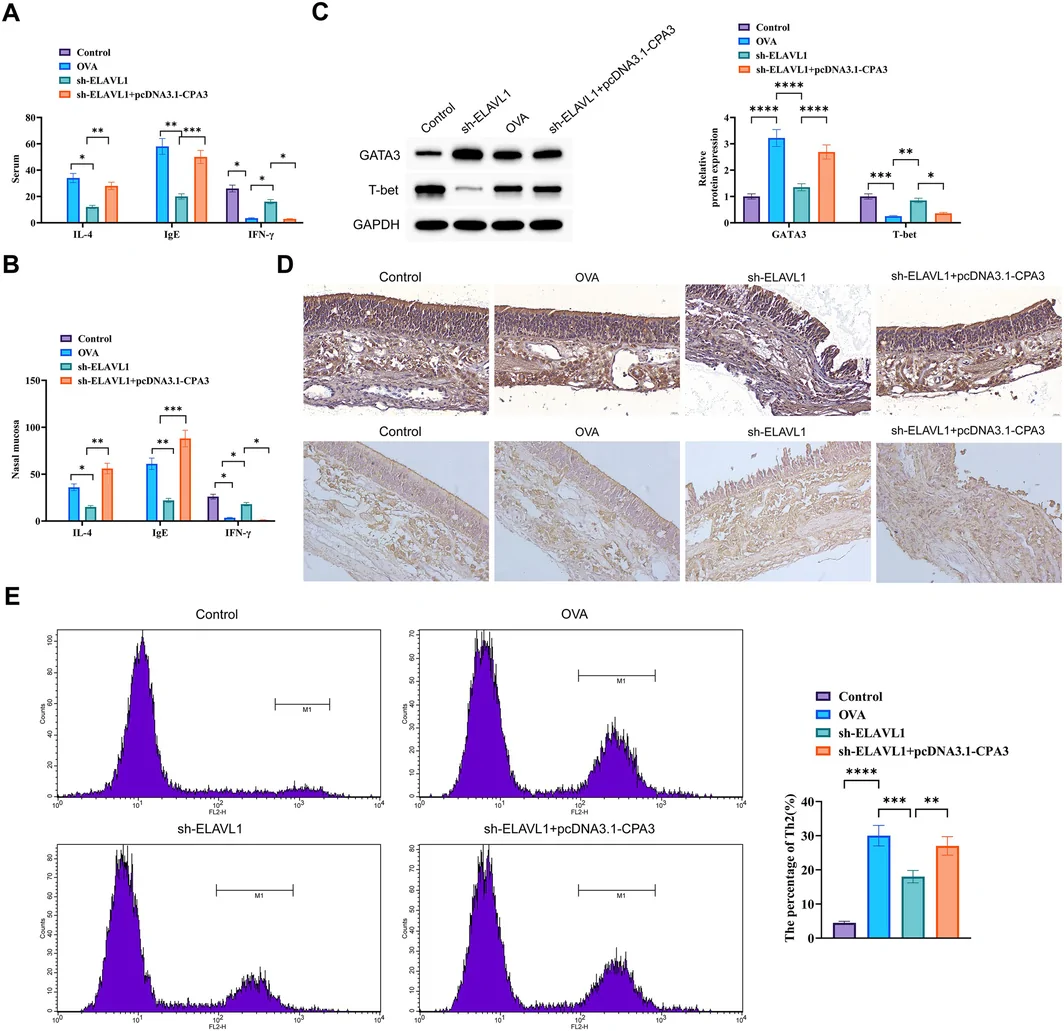
Overexpression of CPA3 reverses the inhibitory effect of ELAVL1 knockdown on Th2 differentiation. (A) Serum levels of OVA‐specific IgE, IL‐4, and IFN‐γ, as detected by ELISA. (B) Levels of OVA‐specific IgE, IL‐4, and IFN‐γ in nasal mucosal tissues, as detected by ELISA. (C) Protein expression of T‐bet and GATA3 in mouse nasal mucosal tissues, as detected by Western blot. (D) Expression of T‐bet and GATA3 in mouse nasal mucosal tissues, as detected by IHC. (E) Proportion of Th2 cells, as detected by flow cytometry. Error bars represent at least three independent experiments; data are presented as mean ± SD. Comparisons were made using Student's *t*‐test and one‐way ANOVA. *p < 0.05, **p < 0.01, ***p < 0.001, ****p < 0.0001.

**FIGURE 8:**
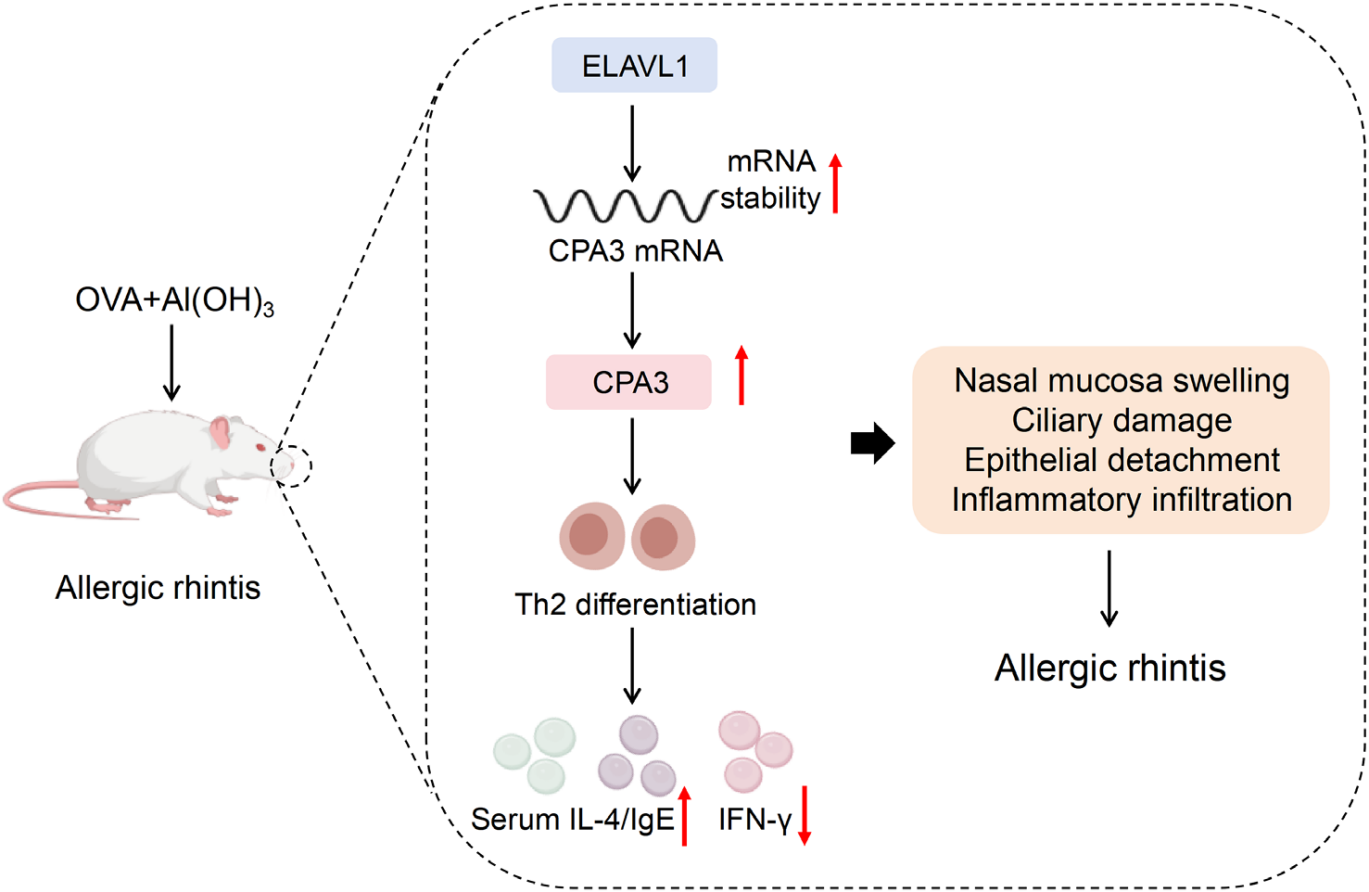
The ELAVL1/CPA3 axis regulates Th2 cell differentiation in allergic rhinitis progression.

To explore the regulatory mechanism between ELAVL1 and CPA3 in allergic rhinitis, the serum concentrations of OVA‐specific IgE, IL‐4, and IFN‐γ were quantified using ELISA. Silencing of ELAVL1 resulted in a significant reduction in OVA‐specific IgE and IL‐4, along with an elevation in IFN‐γ, indicating a shift toward Th1‐type immunity. Conversely, concurrent overexpression of CPA3 counteracted these alterations, leading to increased OVA‐specific IgE and IL‐4 and reduced IFN‐γ levels (Figure 7A). Consistent trends were observed in nasal mucosal tissues, where AR model mice exhibited elevated OVA‐specific IgE and IL‐4 and reduced IFN‐γ. ELAVL1 knockdown attenuated these changes, which were subsequently reversed upon CPA3 overexpression (Figure 7B). Western blot analysis further demonstrated that AR mice showed increased GATA3 and decreased T‐bet expression in nasal mucosa. ELAVL1 knockdown suppressed GATA3 and enhanced T‐bet levels, effects that were negated by CPA3 overexpression (Figure 7C). IHC findings revealed intense GATA3 and faint T‐bet staining in AR nasal tissues. ELAVL1 deficiency reduced GATA3 and intensified T‐bet signals, both of which were restored to AR‐like states by CPA3 overexpression (Figure 7D). Finally, flow cytometric results indicated an expanded Th2 cell population (IL‐4^+^CD4^+^) in AR mice. This proportion was reduced following ELAVL1 knockdown but restored to elevated levels following CPA3 overexpression (Figure 7E). Together, these results suggest that CPA3 acts as a downstream effector through which ELAVL1 promotes Th2 cell differentiation and contributes to AR pathogenesis (Figure 8).

## 4 Discussion

AR is a disease triggered by the immune system's hypersensitivity to environmental allergens. It refers to a noninfectious chronic inflammatory response of the nasal mucosa in susceptible children following exposure to environmental factors such as chemicals and air pollution. As a nonlife‐threatening condition, it often results in low disease awareness among patients. Clinical manifestations include nasal congestion, itching, rhinorrhea, and hyposmia. In severe cases, it can lead to a decreased sense of smell, reduced productivity, and adverse systemic effects [25]. AR has a high global incidence; statistics indicate that the number of affected individuals exceeds 500 million worldwide. The prevalence of AR in China has also gradually increased, from initial regional reports of 11.1% to recent multiregional reports of 17.6%, making AR a growing global health concern [26]. Studies show that the nose and pharynx are key defensive organs of the upper respiratory tract. Secretions from mucosal ciliary cells help prevent the entry of foreign particles and allergens. AR can lead to complications such as pharyngitis and otitis media, causing chronic damage to the middle ear and hearing impairment, significantly affecting children's quality of life [27]. In recent years, the prevalence of AR has shown an increasing trend. Although numerous studies have been conducted, its pathogenesis remains incompletely understood, and current treatments remain suboptimal, necessitating further basic research.

ELAVL1, a well‐characterized RNA‐binding protein, has frequently been reported to exhibit aberrant expression across numerous cancer types [28]. A growing number of studies demonstrate that this protein participates in various cellular processes associated with tumor biology, such as proliferation, cell cycle progression, invasion, migration, and apoptosis [29]. In this study, it was further confirmed that ELAVL1 expression is significantly upregulated in the nasal mucosal tissues of AR patients and is positively correlated with CPA3 and Th2 inflammatory factors, suggesting a key regulatory role for ELAVL1 in the pathogenesis of AR. It has also been reported that immune infiltration analysis indicates associations of ELAVL1, CREB5, CBR1, and NR1D2 with immune cells, suggesting their potential as targets in asthma [30]. Furthermore, ELAVL1 may regulate genes involved in several canonical pathways of T‐cell activation, particularly the CD28 family signaling pathway. These data provide insights into the potential role of Hu antigen R (HuR) in regulating genes and immune mechanisms during T‐cell activation [31]. Targeted inhibition of ELAVL1 impairs Th2‐type inflammatory responses in CD4^+^ T cells in asthma [17], suggesting an important molecular mechanism through which ELAVL1 modulates immune responses.

Through in vitro experiments, including shRNA‐mediated knockdown of ELAVL1 and overexpression treatments, it was demonstrated that ELAVL1 influences AR progression: knockdown of ELAVL1 alleviated cellular inflammation, reduced the apoptotic rate of CD4^+^ T cells, and decreased Th2 cytokine release, whereas overexpression exacerbated inflammatory responses. In vivo mouse models further confirmed that ELAVL1 knockdown alleviated OVA‐induced nasal allergic symptoms, such as reduced scratching and sneezing frequency, as well as improved histopathological injury. Mechanistically, ELAVL1 upregulates CPA3 expression by enhancing its mRNA stability. Functional rescue experiments, in which CPA3 was overexpressed, directly reversed the protective effects of ELAVL1 knockdown, confirming the ELAVL1–CPA3 axis as a key regulatory pathway in AR. This study is the first to elucidate the molecular mechanism by which ELAVL1, as an RNA‐binding protein, promotes AR development by stabilizing CPA3 mRNA, providing a new perspective for targeted therapy. Current AR research primarily focuses on Th2 inflammatory responses and epithelial barrier dysfunction. As an allergic disease, AR is characterized by Th1/Th2 imbalance, eosinophil infiltration, and elevated IgE levels [32, 33]. CPA3, a mast cell‐specific carboxypeptidase, has been shown to be upregulated in asthma and food allergies and is involved in mast cell degranulation and inflammatory mediator release [34], but its role in AR had not been fully elucidated. Similarly, ELAVL1 (HuR), an RNA‐binding protein known to regulate mRNA stability in inflammation and cancer, had not been specifically studied in AR. This study integrates bioinformatics, clinical samples, and experimental models to address this gap, revealing that CPA3 is not only a biomarker but also a downstream effector molecule regulated by ELAVL1. This finding aligns with recent trends emphasizing the role of epithelial‐derived factors in initiating Th2 inflammation [35]. The ELAVL1–CPA3 axis adds a new dimension to epithelial‐immune crosstalk.

A major strength of this study lies in its multilevel and comprehensive experimental design. Complementary methodologies were employed throughout all stages—from clinical discoveries (DEG screening and validation in clinical samples) to mechanistic exploration (in vitro and in vivo knockdown/overexpression models), and further to validation of molecular interactions (RIP, RNA pull‐down, and immunofluorescence co‐localization)—to ensure robust conclusions. For instance, detailed in vitro data from ELISA quantitatively documented the effects of ELAVL1 on cell inflammatory cytokine secretion, while in vivo behavioral tests, H&E staining, and IHC in mice provided clear pathological evidence. The functional rescue experiments, in which CPA3 overexpression reversed the effects of ELAVL1 knockdown, represent a highlight of this study. They not only confirmed the importance of the ELAVL1–CPA3 axis but also highlighted its potential as a therapeutic target. Furthermore, the integration of single‐molecule level mechanisms (such as ELAVL1 enhancing CPA3 mRNA stability) with overall pathological progression is relatively uncommon in previous AR research. Quantitative analysis of Th1/Th2 balance (e.g., flow cytometry detection of IL‐4^+^CD4^+^/IFN‐γ^+^CD4^+^ ratios) further strengthened the evidence for ELAVL1's central role in immune regulation, which is consistent with the immune remodeling mechanism in AR reported by Sun et al. [36]. The findings of this study show both significant consistency with and innovation beyond previous research. In terms of consistency: first, the upregulation of CPA3 in AR aligns with reports by Yan et al., who identified CPA3 as a biomarker in allergic diseases [22]; second, the core pathological features of Th1/Th2 imbalance and eosinophil infiltration are consistent with Zhou et al. [37]; finally, the involvement of ELAVL1 in inflammatory regulation is also supported by Herjan et al.'s research on HuR in airway inflammation [38].

The limitations and future directions of this study warrant discussion. On the one hand, the research primarily relied on mouse models and cell experiments, and the efficacy of ELAVL1 inhibitors has not yet been validated in human clinical trials. On the other hand, although the CPA3 overexpression rescue experiments strengthened the mechanistic link, the specific downstream effector molecules of CPA3 require further investigation. Future studies could explore: the feasibility of specific ELAVL1 inhibitors for AR treatment; the relationship between CPA3‐mediated epithelial damage and mast cell activation; and the generalizability of the ELAVL1–CPA3 axis in other allergic diseases. It is worth noting that CPA3 is mainly expressed in mast cells, whereas the nasal mucosa contains multiple heterogeneous cell types; therefore, the mechanism proposed in this study may reflect intercellular communication among different cell types rather than molecular events within a single cell. Future studies could employ single‐cell sequencing or cell‐specific knockout mouse models to further dissect the regulatory network of this signaling axis at single‐cell resolution. Additionally, the findings in HEK293 cells provide molecular‐level mechanistic evidence, whereas the specific physiological function of this mechanism in the native nasal mucosal microenvironment awaits further validation using primary cells or animal models. In summary, through rigorous experimental logic and novel mechanistic insights, this study establishes the important role of the ELAVL1–CPA3 axis in AR and lays a theoretical foundation for developing targeted therapeutic strategies.

## Ethics Statement

The present study was approved by the Ethics Committee of the Children's Hospital of Chongqing Medical University (Approval No. 202205CQ16) and written informed consent was provided by all patients' parents or guardians prior to the study start. All procedures were performed in accordance with the ethical standards of the Institutional Review Board and the Declaration of Helsinki, and its later amendments or comparable ethical standards. All animal experiments complied with the ARRIVE guidelines and were performed in accordance with the National Institutes of Health Guide for the Care and Use of Laboratory Animals. The experiments were approved by the Institutional Animal Care and Use Committee of Children's Hospital of Chongqing Medical University (Approval No. 202212‐CQ06).

## Conflicts of Interest

The authors declare no conflicts of interest.

## Data Availability

The data that support the findings of this study are available on request from the corresponding author. The data are not publicly available due to privacy or ethical restrictions.
